# Effects of Selenium as a Dietary Source on Performance, Inflammation, Cell Damage, and Reproduction of Livestock Induced by Heat Stress: A Review

**DOI:** 10.3389/fimmu.2021.820853

**Published:** 2022-01-18

**Authors:** Yuhui Zheng, Tian Xie, Shengli Li, Wei Wang, Yajing Wang, Zhijun Cao, Hongjian Yang

**Affiliations:** State Key Laboratory of Animal Nutrition, College of Animal Science and Technology, China Agricultural University, Beijing, China

**Keywords:** selenium, heat stress, gastrointestinal microbiome, inflammation, immunity, antioxidant capacity

## Abstract

Heat stress as a result of global warming has harmful consequences for livestock and is thus becoming an urgent issue for animal husbandry worldwide. Ruminants, growing pigs, and poultry are very susceptible to heat stress because of their fast growth, rapid metabolism, high production levels, and sensitivity to temperature. Heat stress compromises the efficiency of animal husbandry by affecting performance, gastrointestinal health, reproductive physiology, and causing cell damage. Selenium (Se) is an essential nutritional trace element for livestock production, which acts as a structural component in at least 25 selenoproteins (SELs); it is involved in thyroid hormone synthesis, and plays a key role in the antioxidant defense system. Dietary Se supplementation has been confirmed to support gastrointestinal health, production performance, and reproductive physiology under conditions of heat stress. The underlying mechanisms include the regulation of nutrient digestibility influenced by gastrointestinal microorganisms, antioxidant status, and immunocompetence. Moreover, heat stress damage to the gastrointestinal and mammary barrier is closely related to cell physiological functions, such as the fluidity and stability of cellular membranes, and the inhibition of receptors as well as transmembrane transport protein function. Se also plays an important role in inhibiting cell apoptosis and reducing cell inflammatory response induced by heat stress. This review highlights the progress of research regarding the dietary supplementation of Se in the mitigation of heat stress, addressing its mechanism and explaining the effect of Se on cell damage caused by heat stress, in order to provide a theoretical reference for the use of Se to mitigate heat stress in livestock.

## Introduction

**Figure 1:**
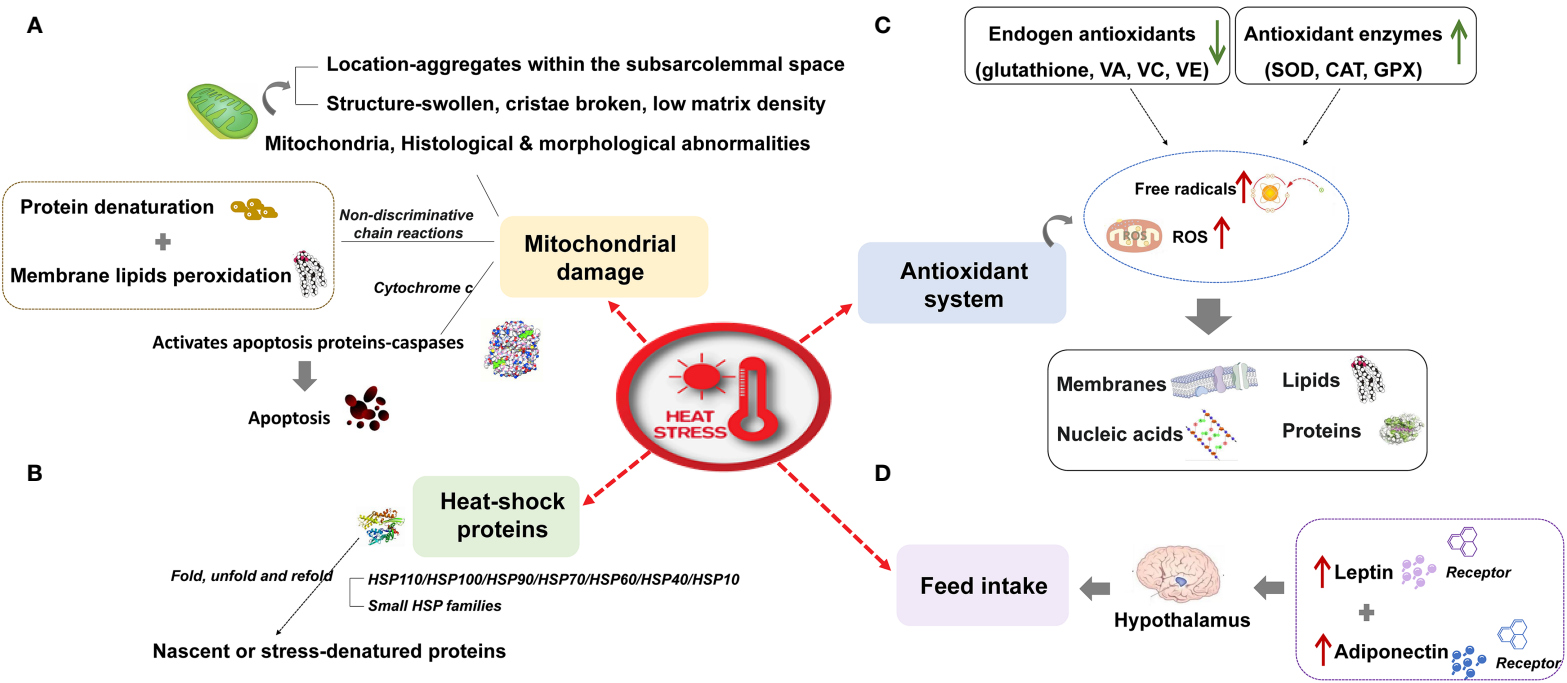
The effect of heat stress on livestock and its molecular response. **(A)** Mitochondrial damage **(B)** Heat shock proteins **(C)** Antioxidant system **(D)** Feed intake.

Ruminants, pigs, and poultry (hereafter grouped as livestock) are extremely susceptible to high temperatures owing to their fast growth rate, fast metabolism, high yields, and sensitivity to temperature. Heat stress caused by global warming has attracted much attention from researchers owing to its harmful effects on livestock, especially high-yielding animals. Heat stress refers to the physiological conditions when the core body temperature of a specific species exceeds the range stipulated by its normal activities. It is caused by the total heat load (internal production and environment) exceeding the heat dissipation capacity (1). Heat stress affects feed intake, the antioxidant system, mitochondrial function, and heat shock protein expression; it disrupts the body’s free radical homeostasis and reorganizes the use of protein, fat, and energy; it subsequently affects animal production, reproduction, and health. The effect of heat stress on livestock and its molecular response are as follows: (I) Inhibition of feed intake: the energy requirements of animals increase under heat stress, but heat stress stimulates the hypothalamus to inhibit feed intake of animals by up-regulating the expression of leptin, adiponectin, and their receptors (2, 3). (II) Damage to mitochondria: heat stress can also cause histological and morphological damage of mitochondria (4), induce fat and protein degeneration (5), and activate the apoptosis pathway based on the release of cytochrome C (6), which intensifies heat stress damage to the body. (III) Oxidative stress: the excessive production of free radicals and reactive oxygen species caused by heat stress (7) can damage the body’s proteins (8), lipids (9), polysaccharides (10), and deoxyribonucleic acid (DNA) (11), and then induce the body to maintain a concentration of reactive oxygen species (ROS) by mobilizing endogenous antioxidants (12, 13) and increasing the activity of antioxidant enzymes (14). (IV) Heat shock protein: the expression of heat shock protein induced by heat stress is a repair mechanism for cells to cope with stress, which can prevent the loss of normal protein function induced by the interaction of denatured protein with neighboring proteins (**Figure 1**).

**Figure 2:**
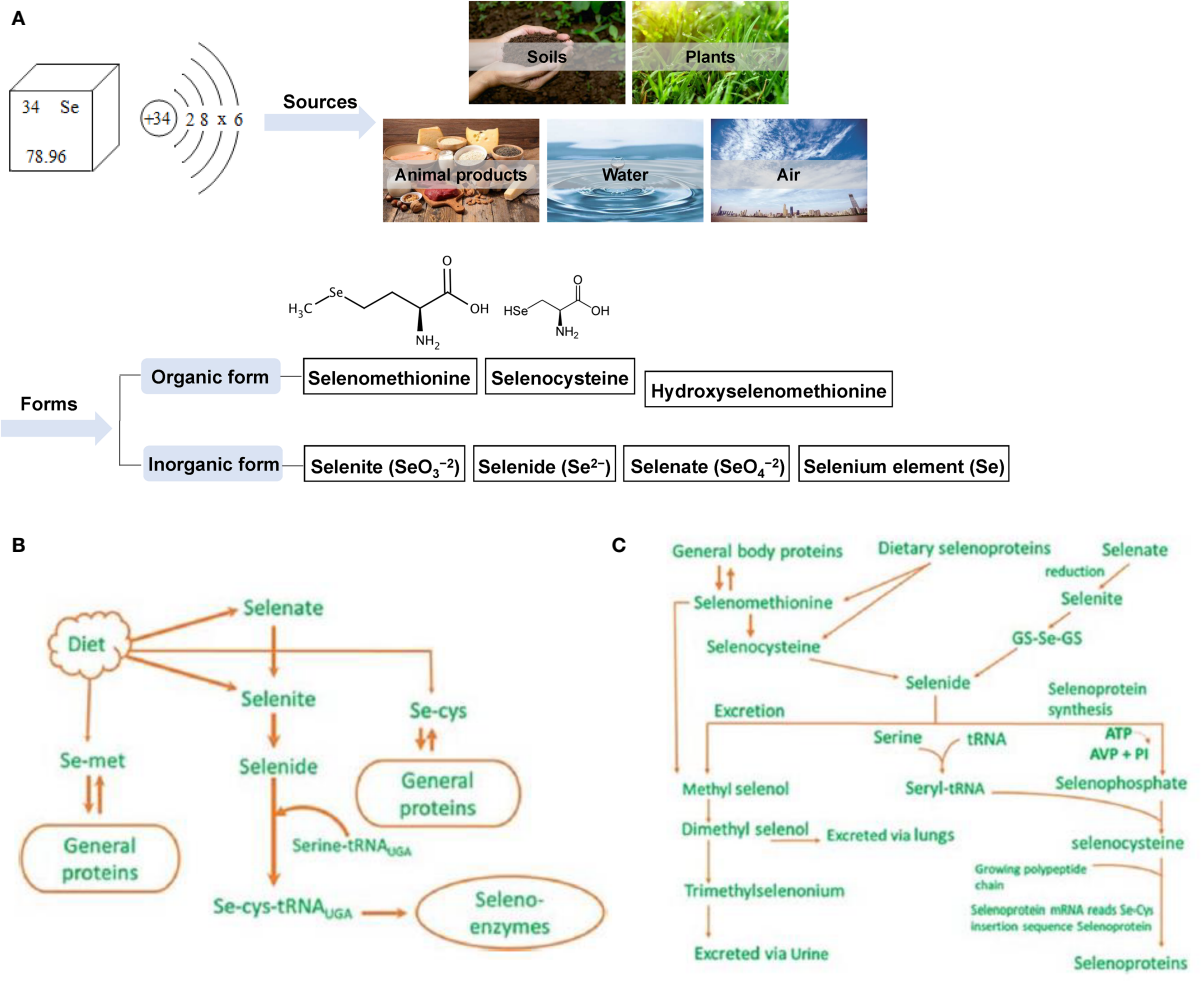
Sources and forms of selenium in nature and its metabolism and excretion in animals. **(A)** The source and form of selenium in nature **(B)** Metabolism diagram of selenium in animals **(C)** Metabolism and excretion of selenium in animals.

Selenium (Se) is an essential nutrient trace element for animal husbandry. It belongs to the same family as oxygen and sulfur and can be combined with a variety of elements to form compounds that are very similar to sulfide. Se exists in nature and organisms in organic and inorganic forms; soil (0.1–0.7 mg/kg), plants (0.02–0.40 mg/kg) (15), animal food sources (0.03–0.34 mg/kg) (16), water (generally ≤10 mg/L) (17), and air (1–10 ng/m^3^) (18, 19) all contain trace amounts of Se. The main inorganic forms of Se include selenite (SeO_3_^2-^), selenate (SeO_4_^2-^), selenide (Se^2-^), and Se (20). Organic forms include selenomethionine (SeMet), selenocysteine (SeCys), and hydroxy-4-methylselenobutyric acid, a new type of organic Se with higher bioavailability (21) (**Figure 2A**). Se is mainly absorbed in the duodenum and cecum of livestock, and its absorption efficiency in ruminants is much lower than that of monogastric animals (22). Dietary protein, vitamin E (VE), and vitamin A (VA) can enhance Se absorption, while diets rich in carbohydrates or nitrates, sulfates, calcium, arsenic, vitamin C, mercury, or hydrogen cyanide can affect absorption (23). Se is stored in different organs and tissues in the form of SeMet in animals as follows: liver 30%, muscle 30%, kidney 15%, plasma 10% and other organs 15% (24). Se is mainly excreted in urine in monogastric animals, while in ruminants, owing to its low intestinal absorption rate, it is mainly excreted in feces (22). The specific metabolism and excretion pathways of Se in animals are shown in **Figures 2B, C** (25, 26). Studies have confirmed that Se can stimulate the formation of antibodies (27, 28), enhance the production of neutrophil chemokines (29), prevent cancer (30, 31) and cardiovascular diseases (32, 33), and enhance animal reproduction (34–36). In addition, the biological functions of Se are mainly mediated by the protein selenoprotein (SEL) containing SeCys, which is the main structural element of SELs such as glutathione peroxidase, thioredoxin reductase, and deiodinase (37, 38). Currently, 30 SELs have been identified in 25 mammalian genes, and they all play a key role in biological functions such as antioxidant, thyroid hormone synthesis, reproduction, and DNA synthesis (17).

## Dietary Supplementation of Se to Improve the Performance of Livestock Under Heat Stress

In tropical, subtropical and arid regions, high-temperature environments have become the main environmental factor affecting animal production (26). As the frequency, intensity, and duration of extreme weather events increases, a rising trend of global temperature has been noted. It is estimated that by 2100, the average global surface temperature will have risen by about 3.7°C (39). Heat stress can severely affect the production and quality of meat, eggs, and milk, as well as the metabolism and health of livestock (40–42), and can even lead to fatalities (14, 43), causing serious economic loss to the livestock industry. In the USA, for example, the annual economic loss caused by heat stress is as high as US$1.2 billion (of which, the dairy industry accounts for US$900 million, and the beef cattle and pigs around US$300 million) (44).

### Growth Performance

Heat stress has an adverse effect on the food intake, digestion, and growth performance of livestock (45, 46). Studies indicate that dietary Se supplementation can significantly reduce the negative impact of heat stress on the growth performance of broilers (47–53). Dietary supplementation of VE and Se can reduce the adverse effects of high ambient temperature on the growth performance of Japanese quail (54), and a combination of 250 mg VE and 0.2 mg Se can maximize their growth performance (55). Supplementing nano-Se in the diet of rabbits suffering severe heat stress can significantly improve their growth performance (56, 57), and dietary supplementation of Se and VE can significantly alleviate weight loss in heat-stressed sheep and improve their feed conversion efficiency (58). Such effects are owing to the following factors: (I) Se can improve feed utilization by regulating the metabolism of carbohydrates, lipids, and proteins (59); (II) Se can improve the antioxidant status of animals, reduce oxidative stress induced by heat stress, and reduce the inflammatory response, thereby promoting growth performance; (III) Se can enhance the ability of livestock to regulate their body temperature (for example, dietary Se significantly inhibits rectal temperature increase in sheep (60), lactating cows (61), and growing pigs (62) affected by heat stress; (IV) Se can maintain and improve growth performance by reducing the adverse effects of heat stress on skeletal muscle (63).

### Production Performance

Heat stress leads to energy balance and metabolic disorders, resulting in a decline in the yield and quality of livestock products (64–67). Studies have found that heat stress reduces egg weight, eggshell thickness, egg yolk index, and egg quality (65, 68, 69). Rozenboim et al. (70) found that supplementing Se to poultry under heat-neutral conditions can increase their egg production and Huff units, and that the supplement SeMet has a stronger protective effect than Na_2_SeO_3_ in reducing oxidative stress caused by heat stress in poultry (71). In addition, dietary Se supplementation could effectively alleviate decreased meat production and the deterioration of meat quality caused by chronic heat stress (72, 73). This is because Se can regulate thyroid hormone metabolism, DNA synthesis, cellular antioxidant levels and immune system responses (74–76)—reducing the adverse effects of heat stress on the metabolism (77) and meat production of livestock (78–81). Heat stress increases free radical and ROS levels in animals, which induce oxidative stress and metabolic disorders (82), and damage the nutrient content (essential fatty acids) and storage stability (flesh color and lipid oxidation) of meat (83–86). Lipid oxidation leads to the production of volatile secondary lipid oxidation products and lactic acid, and reduces meat quality. Studies indicate that the content of the secondary lipid oxidation product malondialdehyde in heat-stressed broiler breast meat can be increased more than two-fold (45, 84). However, the addition of dietary Se can enhance the oxidative stability of lipids in thigh and breast meat, and 125 mg/kg VE and 0.5 mg/kg Se combined supplement is the most effective lipid oxidation inhibitor (78).

Dietary supplementation of Se to heat-stressed sheep can also increase productivity by reducing lipid oxidation in their meat (87). Dietary Se can effectively alleviate the lipid oxidation induced by heat stress, which may be related to an increase in muscle Se content (88–90). Studies have confirmed that the iron, zinc, and Se content of meat is highly correlated with its oxidizing ability (91), and Se is known to be important for improving poultry health and meat quality. Heat stress increases the excretion of minerals in broilers (92, 93), thereby reducing the content of vitamins (VA and VE) and minerals (e.g., iron, zinc, Se) in their tissues (78, 94), resulting in a decrease in oxidative capacity (91). Dietary Se supplementation can be deposited in muscle tissue, which in turn maintains the oxidative stability of its lipids. In addition, Yang et al. (95) reported that the color of meat depends on myoglobin content, which is reduced after oxidation under heat stress. High ambient temperature will reduce red and yellow coloring and increase the pale color of breast meat in chickens (96). The decrease in value caused by such color changes has resulted in more than a one-billion-dollar loss to the USA meat industry annually (97). An Se-rich probiotic diet has been found to increase the redness and yellowness of broiler breast muscles, and reduces the light color caused by heat stress, so it is a beneficial nutritional supplement for improving meat quality in summer (98).

In dairy farming, the temperature and humidity index (THI) has been widely used to measure the heat stress experienced by dairy cows. The following formula is currently proposed by NRC (99) to calculate THI (THI=[1.8×T_db_+32]–[0.55–0.0055×RH]×[1.8×T_db_-26]; where T_db_ = dry bulb temperature, °C ; RH = Relative humidity, %). Furthermore, it is recognized that when the average daily THI exceeds 68, heat stress will cause a decrease in milk production in dairy cows (100). Zimbelman et al. (100) found that when THI increased from 60 to 80, the milk production of dairy cows decreased linearly (for every increase in THI, milk production decreased by 0.13 kg/d); Bohmanova et al. (101) found that when THI was higher than 74, an increase of 1 THI resulted in a decrease in milk production of 0.3 kg/d; furthermore, West et al. (102) confirmed that when THI increased from 72.1 to 83.6, for each THI increase of 1, milk production decreased by 0.88 kg/d.

Further studies have confirmed that heat stress lowers milk quality, which is mainly reflected in the reduction of milk protein, milk fat, and lactose content (103, 104). Compared with other seasons, milk protein content in summer can be reduced by 6% (103); in a different study of heat stress conditions, the protein content of milk was reduced by 4.8% (105). Compared with spray-cooled cows in the dry period, the milk protein content in the following lactation was significantly lower in heat-stressed cows (106). Moreover, milk fat percentage in summer is low (104, 107, 108), and heat stress during the dry period also reduced lactose production of cows in the following lactation (109, 110). Studies have shown that Se can effectively slow down oxidative stress and inflammation in dairy cows, thereby improving health, reducing morbidity, and promoting milk protein synthesis (111–113). However, whether Se can slow down the effects of heat stress on milk production and milk composition needs to be further studied.

## Dietary Supplementation of Se to Relieve Inflammation and Enhance Immunocompetence of Heat Stress Livestock

The mammalian immune system defends against environmental challenges. Stressors suppress immune system components, thereby enhancing the susceptibility of animals to diseases and inducing inflammatory reactions (114). The negative impact of heat stress on the immune system is mediated by cellular immunity and humoral immunity. Cortisol production during acute stress will stimulate the immune system, and during chronic stress, its secretion is related to immunosuppression (115, 116). Se has been shown to be used by almost all tissues and cell types, including those involved in innate and adaptive immune responses (117–119). By increasing Se intake, cell-mediated and humoral immune responses are enhanced (120, 121). Relevant studies have confirmed that dietary Se supplementation can slow down the inflammatory response induced by heat stress through regulating the immune response, thereby improving livestock production.

### Inflammation Reduction

Damage to gastrointestinal physiology and barrier function under acute stress and other pathological conditions can induce various diseases. The gastrointestinal mucosa is covered by the lamina propria and a single layer of epithelial cells. These epithelial cells are connected by tight junctions to form a barrier that restricts the free entry and exit of materials from the intestinal lumen (122–124). The lamina propria contains immune cells, including eosinophils, neutrophils, macrophages, lymphocytes, and mast cells, which can protect the intestines from microbes and their toxic products (125). However, when livestock are in a hot environment, blood is redistributed from visceral tissues to peripheral tissues to maximize radiant heat (126), so the gastrointestinal tract may experience fever, hypoxia, and even inflammation (127–129). Heat stress damages intestinal tight junctions, resulting in impaired intestinal barrier integrity and increased epithelial permeability, which in turn leads to the entry of bacterial endotoxins that trigger local inflammation and immune responses (125), and induce intestinal barrier dysfunction (130). Moreover, there is evidence that oxidative stress caused by heat or other factors can lead to the accumulation of ROS and reactive nitrogen species, which are important predisposing factors of gastrointestinal diseases such as inflammatory bowel disease, intestinal fibrosis and ulcers, colitis, and colon cancer (122, 131). Study found that organic Se from Se-enriched *Agaricus bisporus* can increase the expression of glutathione peroxidase (GPx) by restoring epithelial ion transport and barrier functions, thereby protecting the gastrointestinal tract of rats from heat-induced oxidative stress (132). Increased dietary Se and VE alleviates the effect of heat stress on the integrity of the porcine jejunum and ileal barrier (133). Furthermore, Se has a protective effect on barrier damage and inflammation caused by heat stress in the jejunum of growing pigs (134). Therefore, Se can effectively alleviate intestinal barrier damage induced by heat stress, and follow-up studies should continue to focus on its impact on the structure and function of the intestinal tract of livestock.

According to reports, chronic heat stress significantly reduces liver weight and affects the body’s oxidation response, immune defense, and metabolism (64, 135, 136). It is known that long-term acute heat stress can cause chronic liver damage (137). Moreover, exposure of aged rats to acute heat stress (40–42°C for 24–48 hours) causes liver damage, increased levels of ROS, and changes intracellular signal transduction (138). Further studies have confirmed that dietary Se supplementation can reduce liver oxidative damage after heat stress in rats. This may be related to the ability of Se to activate liver marker enzymes, liver antioxidant status, and liver stress related genes (for example, antioxidant, inflammation, fibrosis, apoptosis, and heat shock) (139). Heat stress significantly increased the activities of aspartate transaminase, alkaline phosphatase, and lactate dehydrogenase in liver tissues; it also increased the content of malondialdehyde, but significantly reduced the level of serum total protein, superoxide dismutase (SOD), and total resistance. Supplementing with Se-rich probiotics can slow down liver damage induced by heat stress by inhibiting liver oxidation, inflammation, and necrosis in a high temperature environment. Compared with a heat stress group, Se supplementation reduced the expression of liver pro-inflammatory cytokines and nuclear factor kappa-B (NF-κB), and reached levels similar to those of a control group that were not exposed to heat stress (139–142). These findings are consistent with previous reports that Se has anti-inflammatory properties (142, 143), and its anti-inflammatory function may be owing to (I) the presence of specific SELs reducing oxidation-induced inflammatory changes in the liver, such as GPx (143–147); (II) Se can improve immunity by up-regulating the ability of immune active cells to respond to inflammation (148–150).

Wooden breast is a type of degenerative myopathy seen in modern broilers, which decreases the quality of breast meat. Studies have confirmed that dietary supplementation of organic Se can improve broiler meat production and increase carcass integrity, thus reducing the incidence of wooden breast. This effect is most likely to be achieved by simultaneously improving the exogenous and endogenous antioxidant status, reducing oxidative stress, and improving tissue healing processes (151). Moreover, heat stress can induce inflammatory damage to mouse lungs, leading to pulmonary edema and lymphocyte infiltration. Lastly, under heat stress conditions and a low-Se diet, the poultry lung exudes large numbers of inflammatory cells (152, 153), which may indicate that the addition of Se in the diet is an important substance to prevent inflammatory damage to lungs.

### Immunocompetence Enhancement

Under heat stress, an animal’s continuous panting changes its blood pH value, leading to respiratory alkalosis. In addition, changes in blood pH can impair immune function and hormonal activity (154). Heat stress seriously damages the growth performance and immunity of livestock, but dietary Se supplementation has been shown to improve immune response in heat stressed broilers (63, 74); Se also supports the immune systems production of inflammation-related enzymes to kill pathogens (155). As mentioned before, heat stress can lead to oxidative stress, including inflammation (156, 157), the first line defense in all forms of cell damage, leading to removal of cell damage, and initiation of cell repair. However, when an inflammatory response is excessive, it causes damage to the surrounding normal cells. When an animal is subjected to oxidative stress such as heat stress, it manifests as the overexpression of lipopolysaccharide or ROS in the body, which is recognized by Toll-like receptors on the surface of immune cells such as monocytes and macrophages (158). The NF-κB pathway initiates the expression of inflammatory genes and produces interleukin (IL) 1, IL-4, IL-6, tumor necrosis factor-α (TNF-α), and other cytokines, which induce an immune response (159, 160). Additionally, heat stress activates the hypothalamic-pituitary-adrenal axis of livestock, which releases glucocorticoids when activated, inhibits the synthesis and release of cytokines, and disrupts the balance between pro-inflammatory and anti-inflammatory factors. The resulting decreased immunity of livestock causes inflammation and reduced feed intake and growth rate, that lead to economic losses in animal husbandry (114, 161).

According to report, the source and level of Se have no notable effect on the performance of broilers subjected to heat stress, *via* spleen and bursal index, blood biochemical indicators, and antibody response to infectious bursal disease virus (162). However, adding SeMet to the diet can improve the feed efficiency of heat-stressed broilers and enhance cell-mediated immunity and humoral immunity (74). Another study showed that Se supplementation had no notable effect on the performance and relative quality of lymphatic organs, but it did improve the antibody response and blood lipid characteristics of heat-stressed broiler red blood cells (163). That is, heat stress significantly reduced the relative quality of the immune organs of broilers and the primary and secondary antibody responses to their red blood cells, while dietary Se supplementation effectively mitigated the negative impact of heat stress on red blood cell secondary antibody responses (163). In addition, studies have found that when sheep are exposed to heat stress, higher dietary Se levels can enhance antibody titers of red blood cells. Supplementation of Se did not affect serum antibody titer of the anti-Newcastle vaccine in broilers (74), and the antibody titer of serum anti-H5N1 increased with the increase of dietary Se level; furthermore, organic Se had a better effect on antibody titer, indicating that Se supplementation using a specific Se source can improve the immune function of heat-stressed broilers (164).

Under heat stress conditions, corticosteroids released in the blood reduce the number of lymphocytes (165), and the immune system is stimulated to increase the number of heterophile cells, which are the first line of defense against stress damage (166). Leng et al. (167) found that organic Se supplements can enhance poultry immune system function by improving the ability of immune active cells to resist infection. Studies have found that: under heat stress, as the dietary Se level increases, the number of heterophils, monocytes, and eosinophils gradually decrease, and serum total protein and albumin levels gradually increase (168); a 5 mg Se treatment can reduce the rectal temperature of sheep by 0.3°C, reduce weight loss by 4.5%, and increase the number of eosinophils (60); injection of antioxidants containing Se, copper, zinc, manganese, VA, VE, etc. before and after weaning of calves in summer can increase their blood immunoglobulin (IgG, IgM, and IgA) concentrations and serum total white blood cells (neutrophils and monocytes) (169); dietary Se supplementation can increase the number of hemameba and hemoglobin in poultry blood (170); 0.25 ppm Se can significantly improve the growth performance of broiler chickens, promote their immune response and lymphatic organ development, and can also increase their serum antioxidant activity and the ratio of heterophile cells to lymphocytes, and reduce the gene expression of heat shock protein (HSP) 70 (171). Furthermore, heat stress-induced cell damage is usually accompanied by abnormal expression of SEL coding genes and SELs, and Se supplementation mainly reduces cell damage induced by heat stress *via* regulating the expression of SELs; that is, Se restores the expression of most SELs in heat-stressed cells at both mRNA and protein levels; in addition, organic Se has a better effect than inorganic Se.

It is known that Se can improve the immune response by changing the production of certain cytokines in immune cells and enhancing the resistance of immune cells to oxidative stress (172). Se added in the diet alone or in combination with vitamins can alleviate the damage caused by oxidative stress and improve immunity (173). Abdel-Moneim et al. (63) supplemented heat-stressed broilers with Se and the levels of immunoglobulins in broilers were notably increased. Moreover, the study found that the addition of dietary Se prevented the up-regulation of six inflammation-related genes induced by heat stress (IL-6, IL-8, intercellular cell adhesion molecule-1, interferon-β, and inducible nitric oxide synthase-2), reduced the expression of pro-inflammatory cytokines in porcine small intestinal epithelial (IPEC-J2) cells under heat stress, and effectively alleviated the adverse effects of acute heat stress on the expression of TNF-α and IFN-γ, thereby reducing immune dysfunction (174). Compared with inorganic Se, an organic Se group had a lower expression of pro-inflammatory genes and better protection (175). Studies have also found that although dietary Se supplementation can inhibit the expression of TNF-α in heat stressed broilers, it cannot prevent the down-regulation of TNF-α expression in IPEC-J2 cells induced by heat stress; therefore, Se can resist heat stress induced inflammatory damage, but this is not achieved by inhibiting the expression of TNF-α (172).

## Dietary Supplementation of Se to Improve Antioxidant Status of Heat Stress in Livestock

Oxidative stress is one of the important factors leading to animal inflammation and immune disorder. As an antioxidant, the moderating effect of dietary Se on inflammatory response induced by heat stress is closely related to its ability to improve antioxidant status of animals. An imbalance between the production of oxides in the body and the antioxidant defense system is the root cause of oxidative stress (176). The biochemical and physiological reactions related to heat stress will increase the production of free radicals (45, 46). Excess free radicals will interact with protein, carbohydrates, lipids, and cells to destroy their structure and function (177); that is, oxidative stress occurs. Free radicals include lipid peroxides, lipid free radicals, ROS, and reactive nitrogen free radicals. Among them, ROS is produced endogenously by organisms during oxidative metabolism. Under normal circumstances, ROS is an important secondary messenger that affects intracellular signal transduction and redox regulation (178), and there is a balance between its production and antioxidant defense. However, under heat stress, the antioxidant defense of cells is unbalanced, and excessive ROS can cause severe damage to biomolecules (lipids, proteins, and nucleic acids), leading to the destruction of cell membrane fluidity and cell apoptosis (179, 180). Studies have shown that heat stress prevents the increase in ROS production in IPEC-J2 cells, and the redox balance is disrupted to trigger oxidative stress. Supplementing Se in an organic form can reduce ROS levels and thus alleviate oxidative stress (175).

Se mainly functions passes through Se such as GPx, thioredoxin (Trx), TrxR, and SELP. Proteins exert an antioxidant function, and there are 25 types of SELs with important physiological functions. The increase in antioxidant capacity is attributed to the inducible Se-dependent antioxidant enzymes. Se is a component of glutathione peroxidase, which combines with VE to counteract free radicals (181). VE is the main fat-soluble antioxidant found in cell membranes. It plays an important role as a chain-cut lipid antioxidant and free radical scavenger in the membranes of cells and subcellular organs (182). Recent studies have shown that VE supplementation has a beneficial effect on meat quality (183), and its combined use with Se can more effectively improve the antioxidant defense system of cells and tissues (78). Glutathione peroxidase can remove ROS, protect cells from oxidative stress damage (184, 185), and prevent lipid and protein oxidation. Studies have shown that dietary Se supplementation can increase the serum Se content and the activity of GPx in broilers, calves, lactating dairy cows, and other animals (63, 169, 186).

At present, the Se sources that researchers add to the diet mainly include SS, SeMet hydroxy analogs, yeast Se, and nano-Se. Different Se sources can increase the activity of GPx in animals under heat stress (63, 186–188). In ruminants, the efficiency of rumen microorganisms using organic Se is 3.8 to 4-fold greater than that of inorganic Se; it is therefore more conducive for rumen microorganisms to synthesize antioxidant enzymes from organic Se in the diet through redox reactions (189). Sun et al. (186) found that the addition of 0.3 mg/kg DM Se in the form of organic Se to the diets of Holstein dairy cows in mid-lactation allowed them to remain stable under heat stress, while GPx activity in the serum of the cows in a similar inorganic Se group decreased gradually. Furthermore, Trx is a multifunctional acidic protein, which exists in two subtypes of Trx1 and Trx2 in animals; TrxR is a pyridine nucleotide/disulfide oxidoreductase, including two isoenzymes TrxR1 and TrxR2. Research has found that the content of Trx in the culture of Bovine Mammary Epithelial Cells was significantly lower than that of heat-treated cells after adding 1 *μ*M SS (190).

The concentration of oxidative stress biomarkers such as SOD, biological antioxidant potential (BAP), and advanced oxidation protein products (AOPP) can also reflect the degree of cellular damage under heat stress. During heat stress, the accumulation of reactive oxygen metabolite ROM in the body leads to a decrease in plasma BAP. The ratio of the two (ROM : BAP) is defined as the Oxidative Stress Index (OSI). AOPP is a marker of protein oxidation when the body is subjected to heat stress, and it also mediates inflammation. Chauhan et al. (191) fed a diet containing 100 IU VE/kg DM and 1.20 mg Se/kg DM to heat-stressed ewes; the results showed that the serum active oxygen metabolites of the ewes were significantly reduced (114 vs. 85 units/dL; P <0.005), physiological antioxidant potential increased (3688 vs. 3985 μmol/L; P = 0.070), heat stress index (ROM/BAP) decreased by 30%, and there was a downward trend of AOPP (19.4 vs. 18.8 mol/L). However, Liu et al. (62) found that feeding 1.0 ppm yeast Se to sows did not alleviate the decrease in blood BAP affect the increase in AOPP during heat stress, but only increased GPx activity by 13%. The above results suggest that when supplementing Se in livestock diets, we should fully consider whether the background value of Se in the basal diet meets the nutritional needs of experimental animals to determine whether additional Se sources can improve oxidative stress. However, it should be noted that Se and VE have a synergistic effect, therefore supplementing the two together may have a better effect.

Oxidative stress activates the heat shock response (192). Heat shock proteins (HSPs), molecular chaperone proteins expressed by the body under stress, can sense oxidative stress and restore physiological protein conformation during and after such stress. The significant increase in their expression is an adaptive mechanism for cells to respond to oxidative stress. When heat stress occurs, Heat shock transcription factor (HSF) is separated from HSP, and HSF enters the nucleus to induce heat shock elements to regulate gene expression and activate the transcription and translation process of HSP (19). According to molecular weight and amino acid sequence, HSPs can be divided into six families: HSP110, HSP90, HSP70, HSP60, small molecule HSPs (HSP27, HSP33, etc.), and ubiquitin. Most HSPs have molecular chaperone protein activity to prevent misfolded protein aggregation that causes damage to cells and promotes the formation of the correct structure of newly synthesized proteins (193). Supplementing Se can significantly reduce the production of HSPs in cells, alleviate the need of cells for HSP protection under high temperature stress, and alleviate oxidative stress (190).

## Dietary Supplementation of Se to Increase Nutrient Digestibility and Regulate the Gastrointestinal Microbiome of Heat Stressed Livestock

### Feed Intake and Nutrient Digestibility

Loss of livestock performance is mainly owing to energy loss caused by reduced feed intake, consumption of feed of low nutrient content, and temperature regulation. Therefore, maintaining the nutrient concentration required for the health and production of heat-stressed livestock is challenging (194). Heat stress adversely affects the feed intake of broilers (195), pigs (196–198), sheep, and dairy cows (191), and the poor health and feed intake associated with heat stress further negatively affects the yield and quality of livestock products. It has been shown that diets supplemented with Se can alleviate the adverse effects of heat stress on animal feed intake and mortality (199).

The effect of heat stress on feed intake (198, 200) is related to heat damage to the intestinal epithelial cells (201, 202). Thus, the effect of Se on the feed intake of heat-stressed livestock may be closely related to its mitigation of intestinal injury. Studies found that when broilers were exposed to heat stress, the flow of blood and nutrients to their gastrointestinal tract was reduced, which led to intestinal hypoxia, adenosine triphosphate consumption, intracellular acidosis, and oxidative and nitrative stress, resulting in changes in intestinal function and integrity (203). Increased intestinal permeability increases the leakage of lipopolysaccharides to the internal environment, leading to eventual multiple organ failure (203). There are also report indicating that heat stress up-regulates the mRNA and protein expression of HSP70, HSP90, and nuclear factor kappa-B, but reduces epidermal growth factor in the jejunal mucosa of black-bone chickens (204). Damage to the gastrointestinal tract reduces Se absorption, which further leads to Se deficiency. In addition, Se can affect gastrointestinal tissues by regulating the production of inflammatory cytokines and increasing the antioxidant status. Se deficiency can lead to the production of harmful free radicals including oxygen and nitrogen free radicals, and at the same time reduce the antioxidant capacity of the intestinal tract, resulting in oxidative damage to the intestinal tissues of chickens (205). Current research shows that: 1.2 ppm of nano-Se supplements can reduce lipid peroxidation and help broilers maintain intestinal structure under heat stress (206); adding 1–3 ppm sodium selenite (SS) to the diet for 90 days increases the Se concentration and the expression of SEL in the gastrointestinal tract of poultry (207); the addition of 0.4 mg of SS per kilogram of diet can enhance the activity of GPx in the blood and liver of broilers and the activity of thioredoxin in the duodenal mucosa, liver, and kidney (208). Therefore, dietary supplementation of Se can effectively reduce heat stress damage to the gastrointestinal tract of livestock, thereby effectively maintaining feed intake.

The reduction of feed intake under high temperatures is mainly to reduce heat production to adapt to the hot environment (196, 197), and the effect of Se on heat-stressed livestock feed intake may be related to its ability to promote digestion and absorption and improve the digestibility of nutrients. Under heat stress conditions, the reduction of feed intake limits total nutrient intake. Furthermore, in order to prevent heat stroke, livestock must prioritize heat dissipation and survival to combat heat stress over other biological processes such as animal production (67, 209). For example, related studies have shown that accelerated respiration for heat dissipation, increases the synthesis of heat shock proteins to prevent cell damage (133, 210), inflammation (211), and the physiological process of repairing damaged tissues (133)—further increasing the mobilization of a sow’s body reserves and impairing production performance and energy availability, as well as nutrient supply. Studies have shown that adding Se to the diet can effectively improve the gastrointestinal function of livestock and the apparent digestibility of nutrients: Wei et al. (212) confirmed that dietary supplementation of 0.3 mg/kg DM Se can promote rumen fermentation and the apparent digestibility of crude protein (CP), neutral detergent fiber (NDF), acid detergent fiber (ADF), and Se in mid-lactation dairy cows; Hassan et al. (57) found that adding 0.5 mg/kg of Se-enriched spirulina to the diet increased the apparent digestibility of DM, organic matter (OM), CP, ether extract (EE), and nitrogen free extract in heat-stressed rabbits; Alimohamady et al. (213) showed that dietary Se supplementation increased the digestibility of DM, OM, CP, NDF, and ADF in 4 to 5 month old lambs.

Both inorganic Se and organic Se can be added to livestock diets. Inorganic Se is more easily reduced to elemental Se that is difficult for the body to use under acidic conditions; whereas organic Se can directly form microbial protein without being reduced to the intermediate product H_2_Se, which improves utilization efficiency (189). The study by Zhang et al. (214) found that coating the Se source is also a way to improve its utilization, and they found that a 0.3 mg Se/kg inorganic Se coating treatment significantly increased the apparent digestibility of DM, OM, and CP in dairy cows. Blood flow plays an important role in controlling body temperature: although under heat stress, blood flow distribution shifts from internal organs to peripheral capillaries to quickly lower body temperature, decreased visceral blood flow can lead to hypoxia in gastrointestinal tissues. When the body lacks an adequate supply of oxygen (such as during metabolism), oxidative stress occurs (215). Hypoxia in the gastrointestinal tract, especially in the intestinal tissues, increases the permeability to pathogens and related endotoxins that cause oxidative stress damage (216, 217), which disturbs the function of the intestinal immune system, promotes deformation of mucous membranes and villi, and causes intestinal infections, which in turn lead to interruption of the digestion and absorption of nutrients (218, 219). Therefore, dietary Se supplementation to alleviate animal heat stress may be related to its ability to alleviate oxidative stress in the gastrointestinal tract and improve nutrient digestibility.

### Gastrointestinal Microbiome

Growing evidence shows that interaction between hosts and their gastrointestinal microorganisms is involved in mammalian nutrient metabolism, immune homeostasis, and pathogen resistance (220, 221). For monogastric animals, it is reported that heat stress affects the structure and composition of the microbiota for from one week to several months (222–226). Xiong et al. (227) and He et al. (223) found that heat stress can increase the relative abundance of *Proteobacteria*, *Gammaproteobacteria*, *Pseudomonadales*, *Moraxellaceae*, and *Acinetobactae* in the intestines of pigs and ducks; Zhu et al. (226) found that heat stress increases the relative abundance of *Bacteroidetes* in the intestines of laying hens; Shi et al. (228) found that heat stress increased the relative abundance of *Firmicutes*, *Tenericutes*, and *Proteobacteria* in the intestines of broilers, but decreased the relative abundance of *Bacteroidetes* and *Cyanobacteria*; Qu et al. (229) found that heat stress increased the relative abundance of *Oscillospira* and *Clostridium* in murine intestines, but decreased the relative abundance of *Lactobacillus* and *Bacteroides*. Study have showed that the *Lactobacillus* was positively associated with serum total antioxidant, while some other microbial species were found negatively associated, such as *Pseudomonadales* and *Acinetobacter* (230). The increase of *Firmicutes*/*Bacteroidetes* ratio was considered to be a typical characteristic of obesity-driven dysbiosis in humans and animals (231). Thus, heat stress may affect animal health by affecting intestinal microorganism. There are still few studies on the effects of dietary Se supplement on the intestinal microbial ecosystem of heat stressed livestock. Further study could focus on this and provide a basis for moderating the heat stress of animals by regulating gastrointestinal microbes. Se in the intestine can enhance the intestinal environment for microorganisms by reducing local inflammation, and can also change susceptibility to infection caused by specific microorganisms (232). A small number of studies have evaluated the effects of dietary Se supplementation on the intestinal microbiota of fish (233, 234) and mammals. These studies confirmed that dietary Se supplementation has a positive effect on bacterial diversity in the intestine (234), produces an increase in beneficial bacteria number (235, 236), and reduces the frequency of intestinal infections (232).

In ruminants, rumen fermentation parameters are closely related to rumen microbes and can reflect their nutrient utilization. Under heat stress, lactating dairy cows significantly increased the production of lactic acid, decreased the production of total volatile acids and acetic acid, and decreased the pH of the rumen, which inhibited the activity of cellulolytic bacteria, resulting in a relative increase in *Streptococci*, *Enterobacteriaceae*, *Ruminobacter*, *Treponema*, and *Bacteroidaceae* in the rumen (199). Dietary Se can promote the growth of rumen microorganisms and rumen fermentation, and can significantly increase the production of propionic acid and total volatile acids. Previous studies have shown that the relative abundance of rumen bacteria, fungi, cellulose, and amylolytic bacteria (such as *Ruminococcus*, *Fibrobacter*, and *Ruminococcus*) increased after adding sodium selenate to the diet of lactating dairy cows; furthermore, the activity of cellobiase, carboxymethyl cellulase, xylanase, and protease were greatly promoted (214). The supplementation of yeast Se in the diet of sheep can increase the relative abundance of flora associated with rumen carbohydrate and protein metabolism (237). Therefore, dietary supplementation of Se may be related to its regulatory effect on gastrointestinal microbes. Future studies can further explore this hypothesis and clarify the role of Se supplementation in livestock diets and its interaction with gastrointestinal microbiota under heat stress.

## Response of Heat Stress Cells to Se

Research on the cellular effects of heat stress began in the 1970s. Results initially showed that heat stress could induce a variety of abnormalities in cell function, including cell membrane fluidity and stability, inhibition of receptors and transmembrane transporters (14), and even induction of oxidative damage and cell death (238). Under stress conditions, structural lipids such as phosphatidylcholine are hydrolyzed to produce phosphatidic acid (239, 240), and cleavage products such as the non-esterified fatty acid may be re-inserted into different membrane sites—leading to changes in membrane structure and membrane fluidity (241). Studies have confirmed that Se supplementation can protect cells from apoptosis induced by heat stress (175). Se regulates the expression of SELs, which participates in a series of cellular defense reactions, thereby protecting cells from stressors such as protein aggregates, heavy metal ions, heat shock, and oxidative damage (242–244).

### Intestinal Cells

The intestinal epithelium plays an important role in the digestion and absorption of nutrients and the development of immune function (245). Studies have shown that reducing the integrity of the intestinal barrier and increasing intestinal permeability through heat stress endangers the health of livestock and their production performance (127, 236, 246). Heat stress will increase the concentration of intestinal endotoxins and pathogenic bacteria in the portal vein and systemic blood (214), leading to gastrointestinal damage and eventual death from heat exhaustion (247, 248). As mentioned above, Se supplementation can reduce intestinal epithelial cell damage induced by heat stress (55, 74, 249, 250).

Heat stress can damage intestinal barrier function, leading to an increase in permeability, and the concentration of lipopolysaccharides in the portal vein and systemic blood (215). Intestinal epithelial cells are closely bound together by tight junction proteins between cells; the latter regulate the permeability of cells and are essential units in the epithelial barrier. Tight junctions are complex structures composed of more than 50 proteins. Studies on the expression of three tight junction proteins: claudin 1, occludin, and zonula occludens-1 (ZO-1) have shown that supplementation of SeMet reduces the down-regulation of ZO-1 and claudin 1 expression under heat stress conditions. Furthermore, SS supplementation alleviates the down-regulation of claudin 1 expression caused by heat stress (175). claudin 1 is a tightly connected structural skeleton and seals the space between two adjacent epithelial cells (251). ZO-1 is a plaque protein that combines with other proteins to form a scaffold or interacts with specific transmembrane proteins to stabilize them in the cytoplasm (252). Interestingly, the first PDZ structural domain of ZO-1 interacts with claudin 1 protein (253), and a decrease in gene and protein expression indicates an increase in the permeability of the epithelial barrier. Both SS and SeMet supplements effectively slowed down the damage of tight junctions, and SeMet even increased the expression of these two tight junction proteins in IPEC-J2 cells exposed to heat stress (175). In addition, occludin mainly regulates the inter-membrane diffusion and paracellular diffusion of small molecules (254), while SS and SeMet supplementation has no significant effect on the protein expression of occludin in IPEC-J2 cells under heat stress (175).

Cells accumulate HSP70 when undergoing heat stress and increased lipid peroxidation, which may serve as a tissue biomarker of potential damage caused by stress (255). In other words, an increase in HSP70 expression usually indicates an increase in the intensity of heat stress. In addition, HSP70 plays a key role in the process of heat resistance by maintaining cell homeostasis (256, 257) as it can protect cells from endotoxemia, hypoxia, and metabolic stress (258); inhibit the activation of caspase3 to prevent heat stress-induced cells apoptosis (259, 260); and activate protein kinase B (Akt) to promote cell survival (261). It is reported that heat stress significantly increases the expression of HSP70 in rat intestinal epithelial IEC-18 cells, which may play a role in protecting such cells from oxidation and heat damage (262). Study showed that SeMet supplementation promotes the expression of HSP70 mRNA and protein in IPEC-J2 cells under heat stress, indicating that it has a beneficial effect on intestinal epithelial cells, i.e., Se reduces heat stress, so cells do not need to synthesize relatively more HSP70 protein to combat heat damage (175).

Heat stress can damage the integrity of the intestinal epithelial barrier of pigs (210, 217, 263), and its mechanism may involve oxidative stress. Although the intestinal oxidative stress markers are closely related to the duration and intensity of heat stress, their expression in the intestinal tract of heat-stressed rats (264, 265) and pigs (209) is significantly increased. Oxidative stress destroys tight junctions (266) and reduces the viability of epithelial cells (258), so it may play a role in the integrity of the porcine intestinal barrier induced by heat stress. Dietary supplements that can alleviate oxidative stress can prevent heat stress caused by intestinal barrier dysfunction. Studies have shown that the expression of SEL in IPEC-J2 cells is affected by heat stress (161). Heat stress induces the expression of 10 SEL-related genes. These genes play an important role in anti-oxidation by promoting hydrogen peroxide metabolism and regulating the level of intracellular stress (161). Study confirmed that heat stress enhances intestinal oxidative stress and reduces barrier integrity, while high levels of dietary Se and VE can reduce the occurrence of oxidative stress and intestinal leakage (133). Studies have shown that Se yeast supplements enhance the resistance of poultry to oxidative stress and high temperature exposure associated with intestinal bacterial infections. This effect is closely related to the improvement of the body’s redox state after Se supplementation (267). In addition, related studies confirmed that the addition of Se to a cell culture medium of caco-2 significantly increased mRNA expression levels of GPx1, thioredoxin reductase (TrxR) 1, and SelP (268). Moreover, supplementation of hydroxyselenomethionine promotes the protein expression of GPx4, thioredoxin reductase (TXNRD) 1, and SELS and down-regulates the expression of seven inflammation-related genes in jejunal mucosa affected by heat stress (134).

The above results suggest that Se supplementation can enhance antioxidant capacity, and thus mitigate damage to the intestinal epithelial barrier of livestock under heat stress. This information provides a research base for alleviating heat stress induced intestinal injury and improving livestock intestinal health.

### Other Cells

The main function of HSP is to resist the effects of stress on cells (269). Heat shock factor (HSF) 1 leads to the expression of stress-induced genes, and HSP90 is the main defense protein against heat stress (270, 271). Under normal circumstances, HSF1 combines with HSP (usually HSP90). When cells are stimulated, HSP separates from HSF1. HSF1 subsequently enters the nucleus and induces downstream heat shock elements to regulate gene expression (272). Studies have shown that the expression of HSF1 and HSP90 genes in bovine mammary epithelial (MAC-T) cells are notably increased after heat shock. Se pretreatment reversed this effect (190), and Se deficiency also increased the level of HSP in chicken livers (273) and the expression of HSP90 in chicken red blood cells (274).

Studies have found that heat stress can increase the production of ROS, which in turn disturbs the steady state of redox balance, leading to oxidative stress in cells (14). Dietary supplementation with Se can increase the activity of glutathione peroxidase in lactating dairy cows and enhance the ability of the antioxidant system (275). Studies have confirmed that oxidative stress in MAC-T cells after heat shock increases the production of ROS and reduces the activities of SOD and total antioxidant capacity (T-AOC), while Se pretreatment can significantly improve the antioxidant effect of MAC-T cells (190). In addition, heat shock and Se pretreatment can affect the expression of HO-1. The endogenous carbon monoxide produced by HO-1 activates Akt/PKB (protein kinase B). Akt has a negative effect on GSK-3β (glycogen synthase kinase 3β) that activates nuclear factor E2-related factor 2 (Nrf2) (276). Nrf2 is a major transcription factor that regulates the expression of antioxidant proteins. Under oxidative stress, its ubiquitination stops and it translocate into the nucleus where it combines with antioxidant response elements, ultimately activating the defense system (277). TXNRD1 is an intracellular SEL and an isoenzyme that provides one of the main enzyme defense systems for ROS in vascular endothelial cells. Studies have found that different forms of Se tend to activate different genes in the Nrf2-antioxidant pathway of dairy cow arterial endothelial cells: SM pretreatment tends to inhibit the expression of Nrf2, while SS tends to reduce the protein level of TXNRD1 (278).

Studies have found that environmental factors greatly affect cell differentiation (279). In the differentiation of mouse myoblasts, Se supplementation reduces the negative effect of heat stress on the myogenic differentiation of C2C12 cells to a certain extent. This process may be related to the change of SEL expression pattern and the effect of SeMet is superior to that of SS (280). It was shown that heat stress increases the expression of most SEL-encoding genes in myoblast (C2C12) cells (281). Among them: the GPx family can use glutathione to catalyze the reduction of hydrogen peroxide and lipid hydroperoxide (282); iodothyronine deiodinase 2 is located in the endoplasmic reticulum (ER) membrane (283) and is an oxidoreductase that participates in thyroid hormone metabolism by catalyzing the activation of tetraiodothyroxine to triiodothyronine (284); SELS and SELK have similar structural features, and they participate in the ER-related degradation of unfolded and misfolded proteins (285); SELT, known as glycosylated transmembrane protein, may have potential functions related to SELW. Studies have shown that: increasing the expression of SELW can compensate for the knockdown of SELT in mouse fibroblasts (286); SEL15 contains a Cys-rich domain in the N-terminal of the protein, which may play a role in catalyzing isomerization or reduction of disulfide bonds (287); the protein encoded by selenophosphate synthase 2 participates in the biosynthesis of SELs, can catalyze the synthesis of monoselenophosphate, and is the main donor of Se (147). Follow-up studies can focus on the expression of the above SELs and further explore the interaction between Se supplementation and heat stress-induced muscle cell damage.

## Dietary Supplementation of Se to Improve the Reproductive Physiology of Heat Stressed Livestock

### Female Livestock

Heat stress is the main risk factor that affects the reproductive efficiency and production performance of female mammals in summer (14, 288). Existing evidence shows that heat stress can cause abnormal atresia of follicles in the ovary, impaired secretion of ovarian steroid hormones, and even lead to infertility (289). Heat stress can cause a significant increase in body temperature and a decrease in egg production, egg weight, ovarian weight, and follicle number (70). Compared with acute heat stress, the impact of chronic heat stress is relatively weak, but owing to its lengthy duration, it also brings serious economic losses to the livestock industry (137). Granulosa cell apoptosis is an important marker and inducer of follicular atresia, and it plays a vital role in maintaining normal ovarian follicular growth, hormone synthesis, and other physiological functions (290). Heat stress inhibits the proliferation of ovarian granulosa cells and induces their apoptosis, which is closely related to the ovarian dysfunction caused by heat stress in various species (291–293); that is, maintaining normal physiological functions of granulosa cells under heat stress may help prevent or reduce ovarian damage caused by heat stress. In addition, in eukaryotes, the endoplasmic reticulum is an important organelle for the folding, modification, and maturation of new and mature proteins.

Many pathological factors can disturb the balance between the protein load and folding ability of the endoplasmic reticulum, which can trigger endoplasmic reticulum stress (294–296). If homeostasis of the endoplasmic reticulum microenvironment is not restored, severe or continuous endoplasmic reticulum stress will eventually induce cell apoptosis (297). There is accumulating evidence that endoplasmic reticulum stress is related to various pathological reactions caused by reproductive diseases and heat stress-induced cell death. Previous studies have shown that endoplasmic reticulum stress-mediated apoptosis of granulosa cells plays an important role in the progression of follicular atresia through 78-kD glucose-regulated protein (GRP78) and CHOP activation in the goat ovary (298). Owing to its antioxidant function, Se has been widely used to regulate metabolic disorders and reproductive physiological functions, such as effectively protecting certain cells from apoptosis induced by poisons and endoplasmic reticulum stress (35). Studies have confirmed that heat stress can reduce the viability of mouse granulosa cells and increase the expression of caspase3, which induces apoptosis, and key apoptosis-related proteins B-cell lymphoma-2-associated X protein and ER stress activation markers GRP78 and CHOP. Sodium selenite can significantly inhibit the decrease in cell viability induced by chronic heat stress, increase the protein expression levels of apoptosis-related genes and endoplasmic reticulum stress activation markers, and inhibit the decrease in estradiol expression in heat stress-induced granulosa cells (299).

### Male Livestock

For male animals, heat stress can change the structure and weight of the testicles, reduce the number of sperms, reduce sperm quality, and cause abnormal sperm morphology and DNA fragmentation (300). The negative effect of heat stress on male fertility is related to the production of ROS (301). For example, studies have found that acute heat stress increases the oxidative stress and ROS levels of SOD 1 knockout mice, where even exposure to 42 C for 15 minutes can cause damage to sperm (136). Therefore, the use of antioxidants may mitigate the negative impact of heat stress on male fertility. The function of Se in the male reproductive tract is independent of other physiological processes in the body. Se acts on the reproductive organs and participates in the biosynthesis of testosterone and the formation and development of sperm (302). In addition, Se is a component of at least 25 SELs, including glutathione peroxidase and other functional and structural proteins of the testis, epididymis, and sperm (303, 304). For example: GPx1 and GPx3 are expressed and located in epididymal epithelia and sperm to protect epididymal parenchyma and mature sperm from oxidative stress; GPx4 can protect developing sperm from DNA damage caused by oxidative stress, it is also a structural component of the middle mitochondrial sheath of sperm and is an important part of sperm stability and motility (305).

Studies have shown that under heat stress, supplementation of 0.3 mg OSe/kg DM in basal diet for rabbits can improve heat tolerance and health status, thereby significantly improving semen quality and subsequent fertility (306). Moreover, the body’s antioxidant/pro-oxidant balance is considered a key determinant of chicken health, embryonic development, sperm quality, and possible production and reproduction characteristics of poultry (307, 308). Studies have found that supplementing organic Se in the diets of heat-stressed roosters increases the number and vitality of sperm, reduces the mortality of sperm, and enhanced the antioxidant status of seminal plasma, thereby improving the quality of seminal fluid (309). Furthermore, the combination of dietary vitamin E and organic Se has a synergistic effect in reducing lipid peroxidation and improving the antioxidant status of poultry seminal plasma, which is specifically increases the number and vitality of sperm under heat stress conditions and reduces sperm mortality rate (310).

## Conclusion

In this review, we summarized the effect of Se as dietary source on heat stressed livestock, while focusing on the performance, inflammation, reproduction and cell damage, as well as the main regulatory mechanism, that is, regulating gastrointestinal microorganisms, nutrient digestibility, antioxidant status, cell damage, and immune capacity of livestock. Hence, Se supplement could serve as a nutritional strategy to help animals to reduce negative effects on their production performances and health during heat stress period.

## Author Contributions

YZ and TX wrote the article. YW, ZC, HY, WW, and SL reviewed and provided guidance for the manuscript.

## Funding

This work was supported by Guiding Local Science and Technology Development Projects by the Central Government (NO.GKZY20111004) and by China Agriculture Research System of MOF and MARA.

## Conflict of Interest

The authors declare that the research was conducted in the absence of any commercial or financial relationships that could be construed as a potential conflict of interest.

## Publisher’s Note

All claims expressed in this article are solely those of the authors and do not necessarily represent those of their affiliated organizations, or those of the publisher, the editors and the reviewers. Any product that may be evaluated in this article, or claim that may be made by its manufacturer, is not guaranteed or endorsed by the publisher.
